# Supplementary figures and images for: Identification of crucial genes in abdominal aortic aneurysm by WGCNA
Source: PeerJ. 2019 Oct 8;7:e7873. doi: 10.7717/peerj.7873 (PMC6788446; doi:10.7717/peerj.7873)

**A**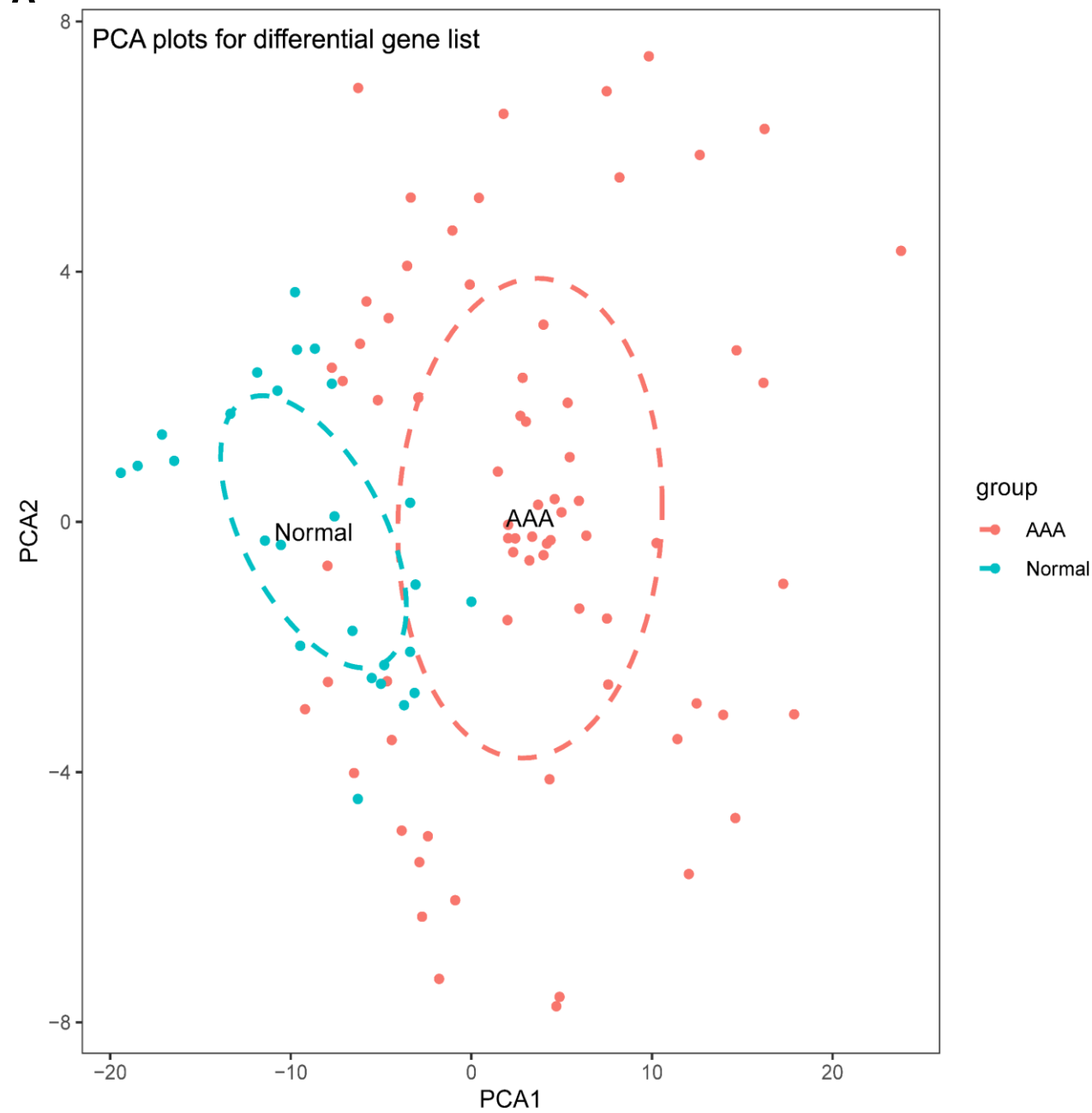**B**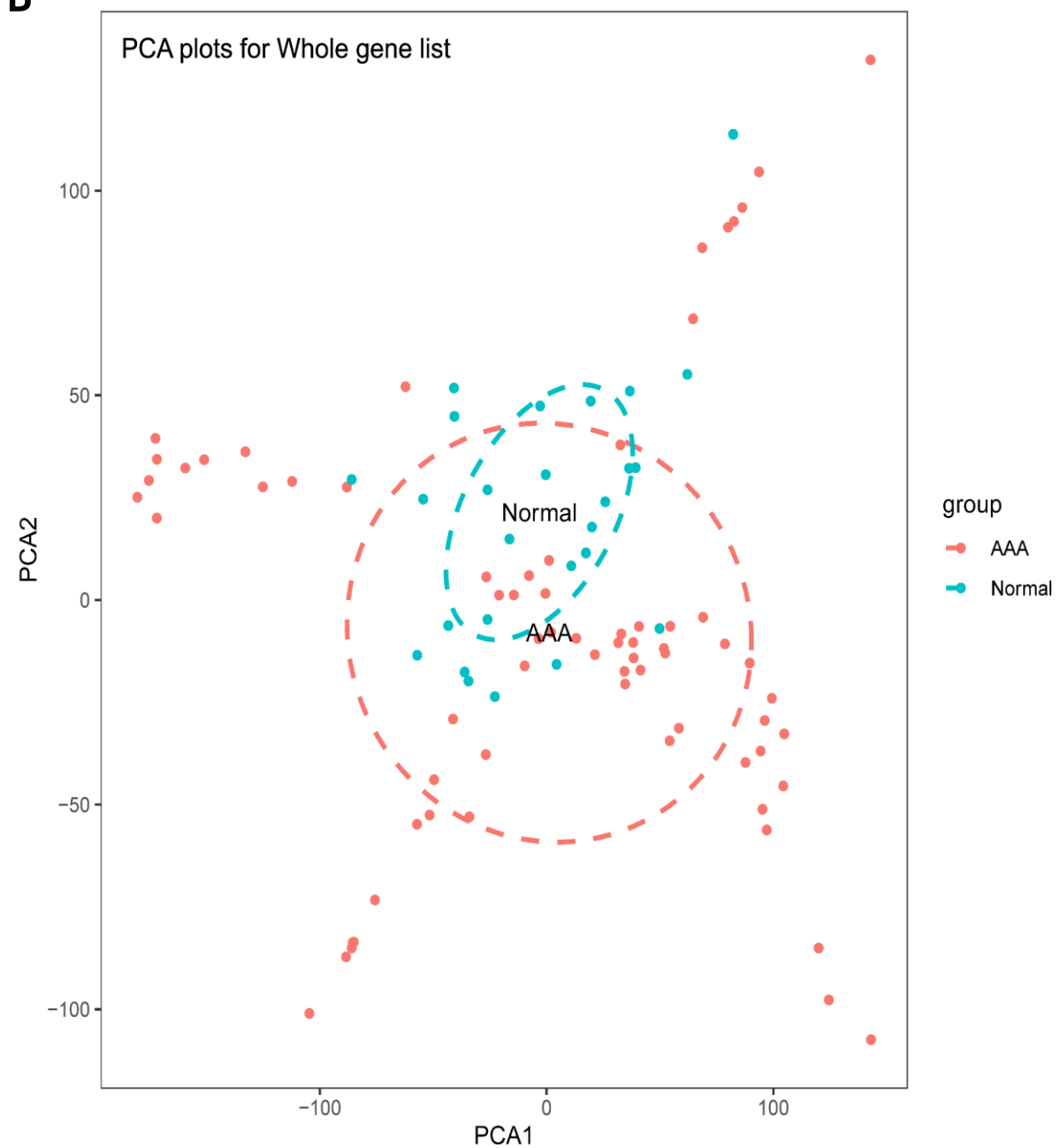

Supplement: Supplemental Information 4 — (A) Based on whole gene list (B) Based on differential gene list. [file peerj-07-7873-s004.pdf]

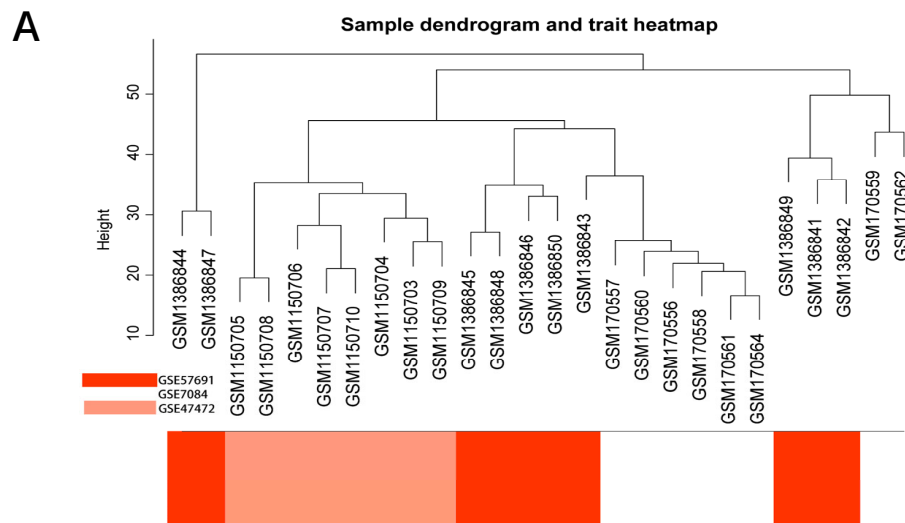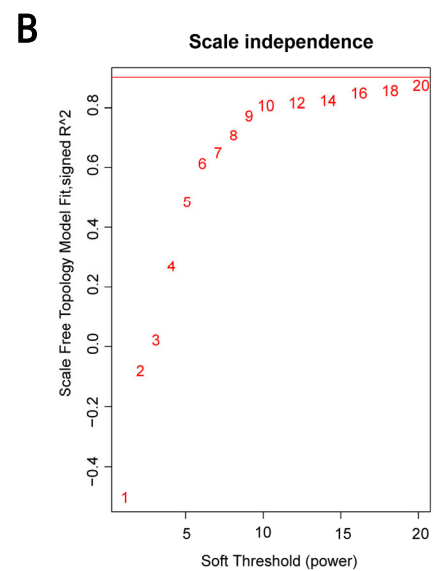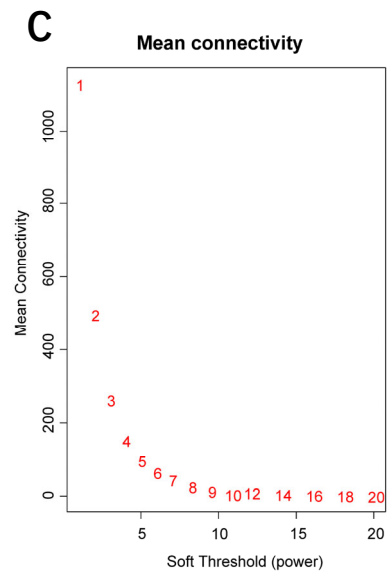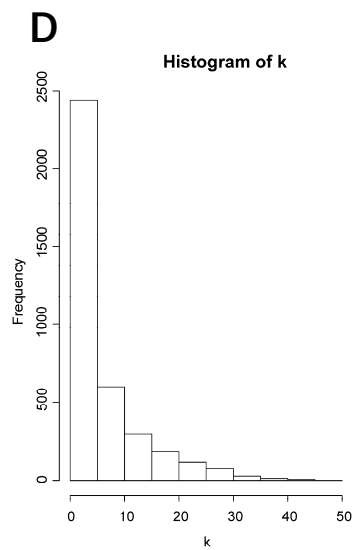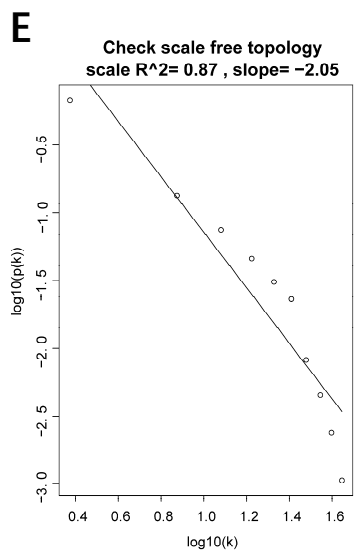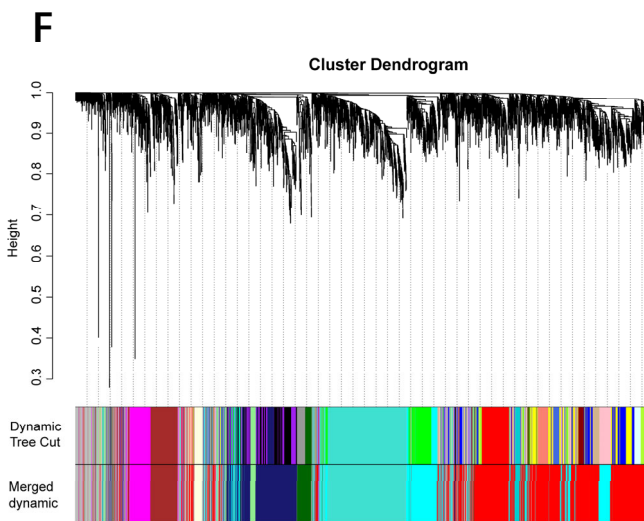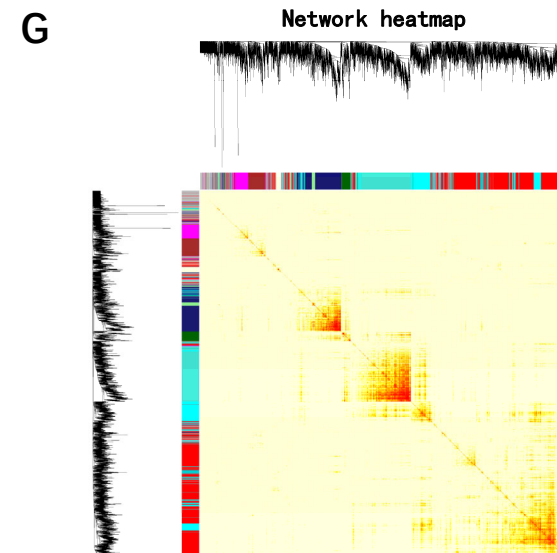

Supplement: Supplemental Information 5 — (A) Sample clustering was conducted to detect outliers. All samples are located in the clusters and pass the cutoff thresholds. (B and C) Soft-thresholding power analysis was used to obtain the scale-free fit index of network topology. (D and E) Scale free topology when soft-thresholding power β = 16. (F) Hierarchical cluster analysis was conducted to detect co-expression clusters with corresponding color assignments. Each color represents a module in the constructed gene co-expression network by WGCNA. (G) Heatmap depicts the Topological Overlap Matrix (TOM) of genes selected for weighted co-expression network analysis. Light color represents lower overlap and red represents higher overlap. [file peerj-07-7873-s005.pdf]
