# Supplementary material for: Identification of crucial genes in abdominal aortic aneurysm by WGCNA
Source: PeerJ. 2019 Oct 8;7:e7873. doi: 10.7717/peerj.7873 (PMC6788446; doi:10.7717/peerj.7873)
Supplement: Supplemental Information 15 [file peerj-07-7873-s015.docx]

We will explain “Related genes” and “All genes” respectively, and black module in AAA condition is presented as one example.

Top 10 KEGG pathways of black module in AAA condition

| Term | Count | Genes |
| --- | --- | --- |
| **Hematopoietic cell lineage** | **14** | **CR2, CD8A, CD3D, CD3E, FCER2, IL7R, CD38, CD37, CD19, CD2, GP1BA, CD4, CD24, CD7** |
| Cytokine-cytokine receptor interaction | 13 | IL2RB, CCR7, CXCR5, CXCR4, CXCL13, IL10RA, TNFRSF13B, TNFRSF13C, TNFRSF17, IL7R, LTB, CD27, LTA |
| **Primary immunodeficiency** | **11** | **CD19, CD3D, CD8A, CD3E, ICOS, TNFRSF13B, TNFRSF13C, CD4, CD79A, IL7R, ADA** |
| Cell adhesion molecules (CAMs) | 11 | HLA-DQB1, ITGAL, CD8A, SELL, ICOS, ITGB7, ICAM3, CD2, CD4, CD6, HLA-DOB |
| HTLV-I infection | 10 | HLA-DQB1, ITGAL, IL2RB, CD3D, CD3E, TNFRSF13C, CDC20, TCF3, HLA-DOB, LTA |
| **B cell receptor signaling pathway** | **9** | **CARD11, CD19, CR2, RAC2, PLCG2, CD79B, INPP5D, CD79A, CD72** |
| **T cell receptor signaling pathway** | **9** | **ITK, CARD11, CD3D, CD8A, FYN, CD3E, ICOS, CD247, CD4** |
| **Intestinal immune network for IgA production** | **8** | **HLA-DQB1, CXCR4, ICOS, ITGB7, TNFRSF13B, TNFRSF13C, TNFRSF17, HLA-DOB** |
| **Natural killer cell mediated cytotoxicity** | **7** | **CD48, ITGAL, SH2D1A, RAC2, FYN, PLCG2, CD247** |
| Chemokine signaling pathway | 7 | ITK, CCR7, CXCR5, RAC2, CXCR4, CXCL13, GNG7 |

**Related genes=Number of genes associated with targeted pathways in KEGG pathways with top 10 count number.**

Black module is associated with immune response, therefore, the targeted pathway in KEGG pathways with top 10 count number means pathways associated with immune response. These pathways are hematopoietic cell lineage, primary immunodeficiency, B cell receptor signaling pathway, T cell receptor signaling pathway, intestinal immune network for IgA production, and natural killer cell mediated cytotoxicity (red and bold letters in the table above). Numbers of genes associated with targeted pathways in KEGG pathways with top 10 count number referrers to the total number of genes of these pathways (not the sum of count number of these pathways because genes enriched in different pathways may be overlapped, for instance, CD3D, CD3E, IL7R, CD38, CD19 and CD4 are both enriched in hematopoietic cell lineage pathway and primary immunodeficiency pathway. Totally, there are 36 genes associated with targeted pathway, therefore, the Related genes=36.

**All genes=Number of all genes in KEGG pathways with top 10 count number**

Numbers of all genes in KEGG pathways with top 10 count number referrers to the total number of genes all the 10 pathways in the table above. Totally, there are 50 genes for all the 10 pathways, therefore, the All genes=50.

The percentage of genes associated with targeted pathways (pathways of immune response for black module in AAA condition) (related genes%) = $\frac{Related genes}{All genes}*100\%.$ Therefore, related genes% =$\frac{36}{50}*100\%$=72%.

Then we found that these targeted pathways were distributed in darkgreen module in normal condition. So we calculated the related genes% for darkgreen module in normal condition using the same method.

Finally, we compared the two percentage using chi-square analysis test and find that related genes% for black module in AAA condition is significant larger than the related genes% for darkgreen module in normal condition (72.0% v.s. 46.7%, p=0.023). Thus, we inferred that the pathways associated with immune response were scattered in normal condition.

Similar analysis was conducted on the red and turquoise modules for the AAA condition and the corresponding modules for the normal condition.
